# Professional Roles, Skills and Advanced Educational Programs of Breast Cancer Nurses: A Scoping Review

**DOI:** 10.1002/nop2.70251

**Published:** 2025-09-28

**Authors:** Lucia D'Alessandro, Sara Morales Palomares, Stefano Mancin, Marco Sguanci, Daniela Cattani, Simone Cosmai, Diego Lopane, Margarita Gjeloshi, Giovanni Cangelosi, Beatrice Mazzoleni

**Affiliations:** ^1^ Department of Biomedical Sciences Humanitas University Pieve Emanuele Milan Italy; ^2^ Department of Pharmacy, Health and Nutritional Sciences (DFSSN) University of Calabria Rende Italy; ^3^ IRCCS Humanitas Research Hospital Rozzano Milan Italy; ^4^ A.O. Polyclinic San Martino Hospital Genova Italy; ^5^ Units of Diabetology, ASUR Marche Fermo Italy

**Keywords:** advanced practice nurse, breast cancer, clinical nurse specialist, nurse practitioner

## Abstract

**Aim:**

To define the role, competencies and clinical focus of specialised breast cancer nurses and to explore post‐basic academic training programmes available in Western countries.

**Design:**

A scoping review was reported following the PRISMA‐ScR framework and aligned with JBI methodology.

**Methods:**

The research question was formulated using the Population, Concept and Context model. Searches were performed across four databases (Medline, Embase, Cochrane Library, CINAHL and Gray Literature) between July and September 2024. Article screening and data extraction were independently performed by two authors. Descriptive analysis was employed to interpret the data.

**Results:**

From an initial 1499 records, 143 studies were selected, with 16 included in the final analysis. The studies, published between 2001 and 2024, evaluated the role of breast cancer nurses in oncology, focusing on their impact on patient care, health outcomes, quality of life, psychological support and breast cancer nurses‐led interventions. Two studies assessed educational programmes, identifying a diverse range of postgraduate degrees and specialised training options tailored to meet the specific needs of this essential nursing role.

**Conclusions:**

Breast cancer nurses are crucial in delivering comprehensive care and improving QoL for breast cancer patients. They contribute to enhancing clinical outcomes through patient education and support. Ongoing professional development and expanded education are essential to meet evolving patient needs.

**Implications for the Profession:**

Breast cancer nurses play a pivotal role in improving patient outcomes by delivering education, psychological support and holistic care. To keep pace with the increasing complexity of breast cancer management, it is essential to prioritise expanded academic training and continuous professional development. These efforts will ensure BCNs are well prepared to address evolving patient needs and provide high‐quality, comprehensive care.

**Patient or Public Contribution:**

No patient or public contribution.

## 1 Introduction

Globally, cancer remains one of the leading causes of death, with breast cancer representing the most common type among women in many regions worldwide (Bray et al. 2024). With 2.3 million new cases, accounting for 11.7% of all cancers, breast cancer has surpassed lung cancer to become the most widespread cancer (Sung et al. 2021). The global increase in breast cancer incidence is linked to both aging and population growth, as well as to changes in major risk factors associated with socioeconomic development (Sung et al. 2021). Advanced breast cancer (ABC) poses a complex and multidimensional challenge in the field of oncology. This condition has greatly benefited from advances in management and treatment, leading to significant improvements in patients' quality of life (QoL) and prolonged survival (Bland et al. 2023; Cloconi et al. 2024). However, the prevalence and incidence of ABC are significantly affected by risk factors related to lifestyle changes, sociocultural norms and environmental factors, which have been profoundly influenced by global economic expansion and the increasing participation of women in the workforce (Chao Shang and Xu 2022).

There are various breast cancer risk factors, including genetic, environmental and behavioural elements. For example, alcohol consumption, overweight and a sedentary lifestyle correlate with increased breast cancer incidence rates (Chao Shang and Xu 2022; Sung et al. 2021). In recent decades, the prevalence of breast cancer risk factors has been significantly influenced by lifestyle changes, sociocultural norms and environmental factors resulting from global economic expansion and increased participation of women in the labour market (Chao Shang and Xu 2022). These changes have contributed to reducing disparities in breast cancer incidence rates between industrialised and developing nations. This is confirmed by recent studies emphasising the persistent burden of breast cancer and the evolving needs in nursing support roles (Ghose et al. 2024; Renouf et al. 2024).

Additionally, hereditary genetic mutations, particularly in the BRCA1, BRCA2 and PALB2 genes, play a crucial role in increasing the likelihood of developing breast cancer (Godet and Gilkes 2017).

The management of breast cancer requires the involvement of a multidisciplinary team of experts, including oncologists, surgeons and radiotherapists. Early diagnosis, generally achieved through mammography or physical examination, often makes primary care physicians the first point of contact for patients (Trayes and Cokenakes 2021). The staging of the disease, which is essential for determining the treatment plan, is based on tumour size, lymph node involvement, presence of metastases, and specific biomarkers such as oestrogen receptors, progesterone receptors and the ERBB2 (HER2) receptor (Trayes and Cokenakes 2021).

Breast cancer staging is essential for defining the treatment plan and is determined by considering tumour size, lymph node involvement, metastasis presence and specific biomarkers such as oestrogen receptors, progesterone receptors and the ERBB2 receptor (formerly known as HER2). It has been shown that active patient involvement in their care management leads to better experiences and clinical outcomes, highlighting the importance of patient education and a patient‐centered approach. However, ABC continues to pose a significant challenge, with a 5‐year survival rate of 25%–30% (Grimm et al. 2022). Consequently, addressing the emotional and psychological challenges accompanying the disease is often difficult due to a lack of resources or effective coordination among different healthcare team members.

In this context, the need for specialised professionals who can offer targeted and comprehensive support becomes evident (Brown et al. 2021). Breast cancer nurses (BCNs) can play a key role in bridging these gaps, thanks to their specific expertise in managing various aspects of the disease. This role can significantly improve clinical outcomes and the QoL of patients by providing personalised and coordinated care within a multidisciplinary team (Brown et al. 2021). The active involvement of the specialised nurse in care management, through continuous patient education and a patient‐centered approach, can enhance patient experiences and clinical outcomes. This is particularly crucial in addressing the challenges of ABC, where integrated and coordinated support is essential for improving QoL and survival outcomes.

The primary objective of the review is to identify the role, competencies and clinical areas of BCNs. Building on the importance of a holistic and personalised approach, this study explores the impact of integrated and collaborative care provided by specialised nurses in improving clinical outcomes and QoL for patients. Additionally, it examines the post‐basic academic training pathways available for this profession in Western contexts.

## 2 Methods

### 2.1 Protocol and Registration

Protocol registration: This scoping review followed a prospectively registered protocol on the Open Science Framework, available at: 10.17605/OSF.IO/DU82V. The review was conducted in accordance with the Joanna Briggs Institute (JBI) methodology for scoping reviews (Peters et al. 2020) and was complemented by the Arksey and O'Malley framework (Arksey and O'Malley 2005). Adherence to the Preferred Reporting Items for Systematic Reviews and Meta‐Analyses extension for Scoping Reviews (PRISMA‐ScR) (Tricco et al. 2018) was maintained to enhance the study's rigor. This comprehensive approach provides a robust foundation for systematically mapping the existing literature and ensures transparency in reporting the scoping review findings. As this review involved analysis of previously published literature, ethical approval from an Institutional Ethical Committee (IEC) was not required.

### 2.2 Formulation of Research Question

The research question was formulated using the PCC framework (Peters, Godfrey, et al. 2020), which includes three components: Population (P), Concept (C) and Context (C). Specifically, in this review, we included: P: BCNs; C: role, competencies and educational programmes; C: breast cancer care setting.

### 2.3 Eligibility Criteria

This review applied stringent eligibility criteria based on the PCC framework (Peters, Godfrey, et al. 2020). The inclusion criteria encompass primary studies published in French, English, Italian, Spanish or German. These studies focus on identifying the roles, competencies and clinical areas of specialised BCNs. Conversely, studies not available in full text, as well as books, chapters, conference contributions and research involving health professionals other than nurses, were excluded.

Various nursing roles, including Clinical Nurse Specialists (CNSs), Nurse Practitioners (NPs), nurse consultants and nurse clinicians, may be referred to as BCNs, and this terminology will be used for identification in this review.

### 2.4 Information Sources

Following the JBI framework (Peters et al. 2020; Tricco et al. 2018), a systematic literature search was conducted to identify potentially relevant records across four databases: PubMed (Medline), Embase, Cochrane Central Register of Controlled Trials (CENTRAL) and Cumulative Index to Nursing and Allied Health Literature (CINAHL), between July 2024 and September 2024. All records deemed potentially relevant were imported into EndNote 20 software (available at https://endnote.com/) and managed using Microsoft Excel (Godino 2023).

### 2.5 Search Strategy

The search strategy for this study was developed in three distinct steps. Initially, the search was conducted in two relevant databases, using keywords derived from the database thesaurus, including MeSH terms, along with additional keywords and Boolean operators. The search strings were specifically tailored to the databases consulted. The selected keywords, such as ‘nurs*’, ‘clinical nurse specialist’, ‘breast cancer’ were chosen based on the study's established eligibility criteria. Following this, the search was extended to the remaining selected databases to ensure comprehensive coverage of the available literature. Finally, the reference lists and citations of the selected full‐text articles were examined, following the methodology proposed by Peters, et al. (2020) and Tricco et al. (2018). This final step also included consulting Google Scholar, professional organisation websites or other institutional websites to identify additional relevant records from the Gray Literature, further adding depth and inclusiveness to the review. This systematic approach not only reinforces the rigour of the study but also reflects the iterative nature of the review process. To ensure transparency and reproducibility, the detailed search strings used in this review are provided in Table S1.

### 2.6 Selection of Evidence Sources

The selection of evidence sources adhered to the guidelines outlined in the JBI methodology (Peters et al. 2020; Tricco et al. 2018). Duplicate records were initially removed using EndNote 20 software (available at https://endnote.com/) and managed through Microsoft Excel (Godino 2023), followed by an additional manual removal to ensure an accurate compilation of the literature corpus for subsequent analysis. The screening process involved three stages, each independently conducted by two researchers (LD and SM). Any conflicts that arose were resolved with the involvement of a third author (MS), who did not actively participate in the screening process. In the first stage, which focused on titles and abstracts, articles that were not relevant to the population, concept or context were excluded. In the subsequent screening stage, the full texts of records identified during the title and abstract review were obtained through various methods, including the EndNote retrieval function, internet searches, direct access to the journals where the studies were published, and involving librarians to retrieve full texts that are otherwise inaccessible. The screening of these full‐text articles for eligibility was conducted based on predefined inclusion and exclusion criteria, with the exclusion of inappropriate publication types (e.g., books, chapters, conference papers) and those not aligned with the objectives of this scoping review. In the final phase, a comprehensive review of the references and citations of these full texts was performed to identify additional eligible records. After completing the selection process, the results were detailed using the PRISMA for scoping review (ScR) flow diagram, which outlines all the stages of selection.

### 2.7 Data Extraction

To facilitate data extraction to answer the research question and achieve the scoping review objective, a data‐charting form was developed, guided by JBI scoping review methodology (Peters et al. 2020; Tricco et al. 2018). The process was independently conducted by two researchers (LD and SM) to ensure a robust and unbiased approach. Any discrepancies or uncertainties in the extracted data were thoroughly discussed with a third author (MS) until a consensus was achieved, aiming to enhance reliability and accuracy. Extracted data encompassed various elements, including author details, publication year, country of origin, study design, participant demographics, intervention specifics, competencies of BCN and the evaluation of quality/bias.

The extracted data were initially presented in a data extraction table to catalogue the general characteristics of the included studies. Subsequently, the data were analysed based on the primary objective of the review, focusing on the role, competencies and clinical areas of the specialised BCN.

### 2.8 Data Syntesis

The synthesis of the results was categorised based on the review objective, adhering to a narrative approach outlined by JBI scoping review methodology (Peters, Godfrey, et al. 2020; Tricco et al. 2018). Additionally, the results were subsequently organised into specific tables and figures for enhanced clarity and comprehension.

## 3 Results

**FIGURE 1:**
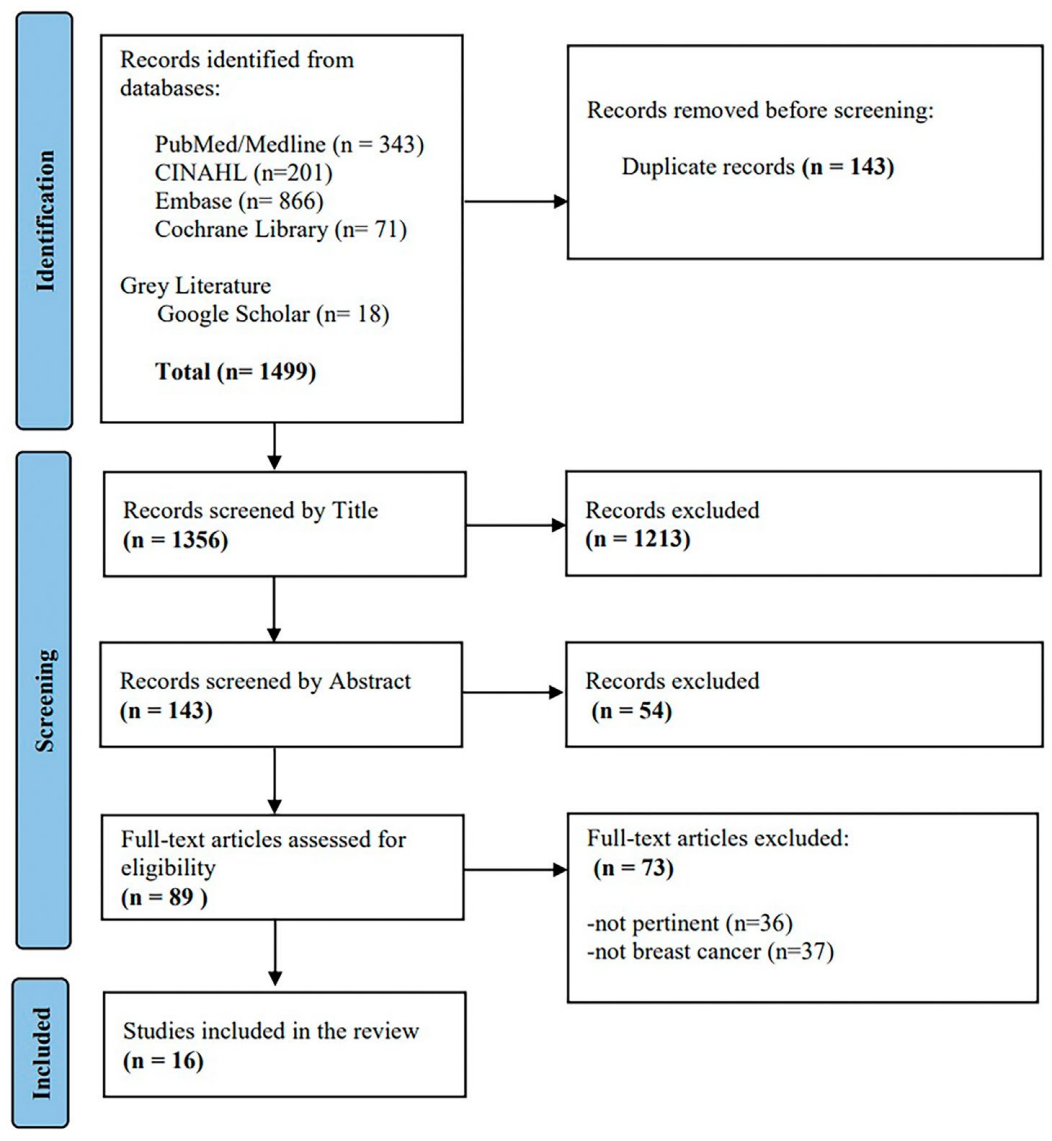
PRISMA‐ScR flowchart of the process of article inclusion.

This scoping review was based on an analysis of 1499 records identified through recognised databases, including PubMed, Cochrane Library, CINAHL and EMBASE, as well as an examination of Grey Literature. After screening titles and abstracts, 143 studies were deemed suitable for further review. The remaining articles, excluded after full‐text examination, either did not address the role of the BCN (*n* = 36) or were not related to breast cancer (*n* = 37). A detailed examination of the full texts ultimately led to the inclusion of 16 articles in the final analysis (Figure 1).

### 3.1 General Characteristics of the Studies Included

This scoping review included 16 records published between 2001 and 2024. These studies covered a wide range of aspects related to the role and impact of BCNs in oncology, evaluating their contributions to patient care, including effects on health outcomes, QoL and psychological support, as well as the feasibility, implementation and acceptability of BCN‐led interventions. Additionally, several studies examined the evolving responsibilities of BCNs in specialised settings, such as advanced breast cancer care, prevention clinics and radiotherapy, as well as the effectiveness of BCNs in meeting patients' informational and emotional needs (Cloconi et al. 2024; Liebert et al. 2003; Vila et al. 2016).

Additionally, two studies (Drury et al. 2023; Ceber et al. 2010) assessed the effectiveness of educational programs designed to improve health professionals' knowledge of breast cancer and to reach consensus on content and pedagogical strategies for online advanced breast cancer educational programs.

Most of the publications originate from the United Kingdom and the United States (Anbari et al. 2023; Brown et al. 2021; Brown et al. 2022; Cruickshank et al. 2020; Droog et al. 2014; Drury et al. 2023; Liebert et al. 2003; Parle et al. 2001; Szwajcer et al. 2004; Vogel 2003). The included articles employed the following study designs: five systematic literature reviews (Brown et al. 2021; Eicher et al. 2006; Hussain Rawther et al. 2020; Rodriguez‐Ortega et al. 2021; Vila et al. 2016), one multicenter project (Liebert et al. 2003), one retrospective survey (Szwajcer et al. 2004), one mixed‐methods study (Cruickshank et al. 2020), two cross‐sectional studies (Droog et al. 2014; Parle et al. 2001), one Delphi study (Drury et al. 2023), one experimental post‐test study (Ceber et al. 2010) and two literature reviews (Cloconi et al. 2024; Vogel 2003).

Our findings indicate that BCNs operate across diverse settings, which can be categorised into hospital‐based and community‐based environments. Hospital‐based settings include acute care hospitals and specialised cancer treatment centres (Cloconi et al. 2024; Liebert et al. 2003; Parle et al. 2001; Szwajcer et al. 2004; Vogel 2003). Community‐based settings, on the other hand, encompass outpatient clinics, primary care facilities and home care services (Rodriguez‐Ortega et al. 2021; Brown et al. 2021; Hussain Rawther et al. 2020; Cruickshank et al. 2020; Vila et al. 2016; Droog et al. 2014; Eicher et al. 2006) (Table 1).

**TABLE 1:** General characteristics of the studies included.

Author, year, country	Setting	Sample	Role	Skills	
Rodriguez‐Ortega et al. (2021) Spain	Hospital/primary health care	NA	Each phase of the disease process	Clinical expertise Coordination of care Education of patients and relatives Emotional support	Managing information and educational needs Support in decision‐making Technical information
Vila et al. (2016) Spain	Hospital/primary health care	NA	Clinical management Nursing education	Prescription and interpretation of diagnostic investigations Education for admission Managing adverse effects and pain Clinical research Teaching (nurses) Communicative abilities	Follow‐up management Education on the management of symptoms Staff training and support Partecipation in education programme Empathy
Liebert et al. (2003) Australia	Hospital	NA	Clinical management	Support in decision‐making Managing information and educational needs Communicative abilities Coordination of care	Education of patients and relatives Education for admission Education on the management of symptoms
Szwajcer et al. (2004) Australia	Hospital	NA	Clinical management Support nursing services	Education for admission Discharge planning Education of patients and relatives Emotional support	Follow‐up management Managing information and educational needs Communicative abilities Technical information
Hussain Rawther et al. (2020) India	Hospital/primary health care	NA	Clinical management Support nursing services Primary care	Clinical expertise Education of patients and relatives Emotional support Empathy Communicative abilities Coordination of care	Managing information and educational needs Support in decision‐making Education on the management of symptoms Managing adverse effects and pain
Brown et al. (2021) UK	Hospital/primary health care	NA	Clinical management Support nursing services Primary care	Follow‐up management Clinical expertise Education of patients and relatives	Communicative abilities Education on the management Managing information and educational needs
Cruickshank et al. (2020) UK	Hospital	NA	Clinical management Support nursing services	Emotional support Empathy Communicative abilities	Follow‐up management Clinical expertise Education of patients and relatives
Parle et al. (2001) Australia	Hospital	NA	Clinical management Support nursing services	Support in decision‐making Education on the management of symptoms	Emotional support Empathy Communicative abilities
Droog et al. (2014) Ireland	Hospital	(*n* = 90) Breast Cancer	Clinical management	Managing information and educational needs Support in decision‐making Education on the management of symptoms	Staff training and support Partecipation in education programme Clinical expertise Coordination of care
Eicher et al. (2006) Germany	Hospital	NA	Clinical management Primary care	Emotional support Empathy Communicative abilities Support in decision‐making Education on the management of symptoms	Technical information Staff training and support Partecipation in education programme Clinical research
Vogel (2003) USA	Hospital	(*n* = 89) Breast Cancer	Primary care	Provide risk education Clinical research Technical information Staff training and support Managing information and educational needs	Communicative abilities Support in decision‐making Education on the management of symptoms
Cloconi et al. (2024) Cyprus	Hospital	NA	Clinical management	Technical information Clinical expertise Coordination of care Education for admission Managing adverse effects and pain	Provide risk education Support in decision‐making Education on the management of symptoms
Drury et al. (2023) UK	Education programmes	NA	Nursing education	Technical information Clinical expertise Clinical research	Staff training and support Partecipation in education programme
Ceber et al. (2010) Turkey	Primary health care	NA	Nursing education	Clinical expertise Clinical research Technical information	Staff training and support Participation in education programme Education on the management of symptoms
Anbari et al. (2023) USA	Primary health care	NA	Clinical management Primary care	Technical information Clinical expertise Coordination of care Communicative abilities	Staff training and support Managing information and educational needs Staff training and support
Brown et al. (2022) UK	Hospital	NA	Clinical management	Technical information Clinical expertise Coordination of care Communicative abilities Empathy Support in decision‐making	Staff training and support Participation in education programme Follow‐up management Managing information and educational needs

Abbreviations: ENT, ear, nose and throat; NA, not applicable; U, unspecified; UK, United Kingdom; USA, United States of America.

To provide a detailed and comprehensive description of the BCN role, the results of this scoping review were organised into three categories: educational programme curricula, roles of BCNs, and effectiveness and evidence of BCN interventions.

### 3.2 Breast Cancer Nurses Educational Programs

#### 3.2.1 Educational Program

One study evaluated the positive impact of an educational program on nurses' knowledge and practices related to breast cancer (Ceber et al. 2010). The program consisted of three components: small group educational presentations, instructional videotapes on performing breast self‐exams, and hands‐on practice using a miniature lump model for breast examination. The experimental group, which participated in this program, demonstrated significantly higher knowledge scores compared to the control group. Additionally, this group showed a higher application rate of mammography and clinical breast examinations, although no significant differences were observed in performing breast self‐exams. Participants in the experimental group also reported greater confidence and motivation, and scored higher on the health belief scale, underscoring the effectiveness of the educational intervention in enhancing breast cancer awareness and early detection practices (Ceber et al. 2010).

#### 3.2.2 Online Educational Programs' Curriculum

The ABC4Nurses program addressed a critical gap in the existing educational standards and competencies for advanced breast cancer care (Dowling et al. 2023). The strength of this initiative lay in its systematic design, grounded in a thorough review of existing evidence and validated through consultation with a broad range of stakeholders. By offering an open‐source, multilingual online platform, ABC4Nurses overcame barriers related to the availability and recognition of specialist education across European countries, enhancing access to essential continuing professional development for cancer nurses, particularly those providing care to individuals with advanced breast cancer. The program focused on seven key areas: understanding the significance of advanced breast cancer, treatment approaches, supportive and palliative care, practical nursing skills, multidisciplinary collaboration, self‐care for nurses, and effective teaching and learning methods (Drury et al. 2023).

#### 3.2.3 Analysis of Educational Programmes in the International Context

An analysis of the educational programmes available for Breast Cancer Nurses (BCNs) across the USA, Canada, Australia, the UK and Europe reveals a diverse range of postgraduate degrees and specialised training options tailored to meet the needs of this essential nursing role (Table 2).

**TABLE 2:** Educational programs and certifications for breast cancer nurses.

Country	Title/course	Training	Certifiers
USA	Master of Science in Nursing (Oncology)	Advanced clinical skills, patient management, research	ONCC
	CBCN	Clinical experience + specialised training	ONCC
	DNP	Leadership, evidence‐based practice, clinical expertise	ANCC
Canada	Master of Nursing (Oncology)	Theoretical knowledge + practical skills in oncology	CNA
	Certified Nurse Specialist in Oncology	Experience in oncology + exam	CNA
	Postgraduate Diploma in Oncology Nursing	Specialised training in cancer care	Provincial nursing regulatory bodies
Australia	Master of Nursing (Oncology)	Evidence‐based practice, advanced clinical competencies	ACN
	Endorsement in Oncology Nursing	Specialised training and proficiency in oncology care	ACN
	Graduate Certificate in Breast Cancer Nursing	Focus on breast cancer management and care	Various universities and professional bodies
UK	MSc in Advanced Practice in Cancer Care	Comprehensive cancer care, clinical assessment	RCN
	Postgraduate Diploma in Oncology Nursing	Specialised oncology training	University‐specific certifications
	NHS Training	Clinical and practical training in breast cancer care	RCN
Europe (only European programs were analysed, not for individual states)	ECON	Standardised competencies in oncology nursing	EONS
	Postgraduate Diploma in Oncology Nursing	Specialised oncology education and practice	University‐specific certifications
	Advanced Oncology Nursing Courses	Advanced clinical skills and research methods	Various European universities and nursing bodies

Abbreviations: ACN, Australian College of Nursing; ANCC, American Nurses Credentialing Center; CBCN, Certified Breast Care Nurse; CNA, Canadian Nurses Association; DNP, Doctor of Nursing Practice; ECON, European Certification in Oncology Nursing; EONS, European Oncology Nursing Society; NHS, National Health Service; ONCC, Oncology Nursing Certification Corporation; RCN, Royal College of Nursing.

##### 3.2.3.1 United States

In the United States, numerous institutions offer specialised programmes for oncology nursing, with a focus on breast cancer care. For instance, the Master of Science in Nursing (MSN) With a specialisation in oncology nursing is available at several American universities. These programmes emphasise advanced clinical skills, patient management and research methods specific to oncology (McGhee and Kearney 2020). Additionally, certification options such as the Certified Breast Care Nurse (CBCN) are offered by the Oncology Nursing Certification Corporation (ONCC), which requires candidates to have clinical experience and complete specialised training (ONCC 2022).

##### 3.2.3.2 Canada

In Canada, institutions such as the University of Toronto and the University of Alberta offer programmes like the Master of Nursing with a specialisation in oncology nursing. These programmes prepare nurses to provide specialised care to breast cancer patients, integrating both theoretical knowledge and practical skills (Wood and Maimon 2019). The Canadian Nurses Association also offers the Certified Nurse Specialist in Oncology (OCN) Certification, which promotes advanced practice in the oncology field.

##### 3.2.3.3 Australia

Australia has made significant strides in oncology nursing education. Programs such as the Master of Nursing (Oncology) at the University of Sydney emphasise evidence‐based practice and advanced clinical competencies in cancer care. The Australian College of Nursing offers the endorsement in oncology nursing, which requires nurses to complete specialised training and demonstrate proficiency in oncology care (ACN 2021).

##### 3.2.3.4 United Kingdom

In the United Kingdom, the educational landscape for BCNs is extensive. Programs such as the Master of Science in Advanced Practice in Cancer Care at institutions like the University of Manchester prepare nurses to manage complex cancer cases, including breast cancer. The curriculum includes modules on advanced clinical assessment, cancer biology and patient‐centered care (Gordon et al. 2020). Additionally, the National Health Service (NHS) supports specialised training programmes for nurses in breast cancer care, ensuring they are equipped with the latest knowledge and skills. The Royal College of Nursing (RCN) also provides resources and professional development opportunities focused on oncology nursing (RCN 2021).

##### 3.2.3.5 Europe

In Europe, the educational landscape for breast cancer nursing is diverse. The European Oncology Nursing Society (EONS) offers the European Certification in Oncology Nursing (ECON), recognised across multiple countries. This certification aims to standardise competencies in oncology nursing, including breast cancer care (EONS 2023). Many universities, such as the University of Edinburgh in the UK, offer Postgraduate Diplomas in Oncology Nursing, emphasising the importance of specialised training in breast cancer care (Thompson and Edwards 2018).

### 3.3 Role of Breast Cancer Nurses

#### 3.3.1 Primary Prevention

BCNs play a vital role in implementing primary prevention strategies for breast cancer, contributing significantly to reducing incidence, morbidity and mortality. Their advanced skills in health assessment and decision‐making enable them to evaluate patients' risk factors, identify relevant physical findings and provide comprehensive education on risk mitigation (Vogel 2003). BCNs also synthesise data to develop recommendations for surveillance, pharmacotherapy, lifestyle interventions and genetic counselling, guiding patients towards personalised preventive measures (Vila et al. 2016). Their ability to manage psychological and psychosocial aspects, such as anxiety and fear of recurrence (FCR), further supports patients in addressing emotional concerns associated with their cancer journey. However, Cruickshank et al. (2020) observe that many BCNs feel limited in their techniques for managing FCR, underscoring the need for targeted training in this area.

The role of BCNs has evolved beyond primary prevention, now encompassing complex care pathways that require multidisciplinary collaboration to improve outcomes, particularly for women with early and advanced breast cancer (Brown et al. 2021). This evolution reflects their expanded capacity not only in preventive care but also in follow‐up and supportive interventions throughout the treatment process. In global contexts, especially in developing countries, there is increasing recognition of the need for BCN‐led initiatives to enhance care and advance breast cancer research (Hussain Rawther et al. 2020). Standardising BCN roles, as emphasised by Eicher et al. (2006), is essential for optimising services and ensuring BCNs effectively address the physical, psychosocial and educational needs of breast cancer patients in both hospital and community settings. Through their involvement in primary prevention, education and psychosocial support, BCNs empower patients to make informed healthcare decisions and actively manage their risk factors. As their role continues to expand, BCNs are set to play an even greater part in oncology care, contributing to patient advocacy and improving patient outcomes across diverse healthcare systems.

#### 3.3.2 Hospital Setting, Outpatients and Primary Care

A study conducted in Australia (Szwajcer et al. 2004) evaluated the responsibilities of BCNs, which include assessing the physical and psychosocial well‐being of women undergoing treatment, providing information to patients and families, and coordinating care across multiple treatment modalities. Patient interactions with BCNs at three stages (pre‐surgery, during hospitalisation and post‐discharge) revealed notable trends. Post‐operative contact was highly valued, with many patients finding guidance on care and recovery beneficial. Follow‐up reassured patients, particularly those discharged early, that they had continued access to the BCN. However, some patients expressed a desire for more contact prior to surgery. Emotional support and discussions about cancer‐related concerns were praised, though areas for improvement in communication were identified (Szwajcer et al. 2004; Liebert et al. 2003). BCNs also conduct comprehensive assessments, develop individualised care plans and implement targeted interventions for patients. They ensure continuity of care across healthcare services, support long‐term survivorship, and provide essential education on treatment options and side‐effect management. By leveraging their advanced clinical skills, BCNs enhance patient outcomes and improve the QoL for individuals managing breast cancer (Anbari et al. 2023).

Another important role of BCNs, highlighted in a recent study (Cloconi et al. 2024), is enhancing the QoL for advanced breast cancer patients undergoing radiotherapy (RT). Their responsibilities encompass direct patient care, side‐effect management, education and counselling. BCNs address the psychological challenges associated with advanced breast cancer, providing support that helps alleviate feelings of isolation. Integrating patient‐reported outcome measures into clinical practice is recommended to personalise care and monitor adverse events. BCNs also play a key role in ensuring adherence to treatment plans, clarifying patient concerns and improving treatment outcomes. As radiation therapy continues to advance, ongoing developments in education and clinical practice are essential to optimise care for advanced breast cancer patients (Cloconi et al. 2024).

#### 3.3.3 Management of Fear Cancer Recurrence

BCNs play a critical role in managing Fear of Cancer Recurrence (FCR), though their approaches and confidence levels can vary significantly (Cruickshank et al. 2020). Vila et al. (2016) emphasise the importance of specialised oncology nurses like BCNs within multidisciplinary teams to provide psychological support, improve care coordination and enhance patient outcomes.

While FCR is widely recognised as a major source of distress, there is no consensus on its prevalence, and many BCNs tend to focus more on physical symptoms than on the psychological dimensions of recurrence. The Mini‐AFTERc intervention shows promise as a tool to address these psychological aspects; however, many BCNs require additional training and assessment tools to effectively manage FCR (Cruickshank et al. 2020).

Rodriguez‐Ortega et al. (2021) further confirm that BCNs improve patient well‐being and satisfaction, though the direct links between BCNs' interventions and survival outcomes remain unclear.

#### 3.3.4 Fertility Preservation

Fertility preservation (FP) is a crucial consideration for young women diagnosed with breast cancer, and BCNs play an essential role in facilitating this aspect of care. BCNs provide emotional support and address fertility concerns by coordinating communication between patients and healthcare providers. Their involvement is particularly valuable, as they often discuss the psychosocial aspects of a cancer diagnosis—an area emphasised by both BCNs and other healthcare professionals (Brown et al. 2022). Through specialised training, BCNs gain expertise in individualised patient care, although knowledge levels may vary among clinicians (Rodriguez‐Ortega et al. 2021). Despite this variability, BCNs are highly regarded for their sensitive approach to FP discussions, often stepping in when breast surgeons and oncologists overlook these conversations (Brown et al. 2022). Indeed, the inclusion of BCNs in multidisciplinary teams has been shown to improve patient satisfaction, continuity of care and the overall coordination of complex care (Vila et al. 2016). Empowering BCNs to lead FP discussions could enhance the timeliness and clarity of information, ensuring that patients' needs are fully addressed, thus contributing to improved experiences and potentially better clinical outcomes (Rodriguez‐Ortega et al. 2021).

#### 3.3.5 Supporting Quality of Life

A key role of BCNs is enhancing the QoL for women with breast cancer, along with improving satisfaction with the nursing care they receive. Evidence consistently shows that BCN‐led interventions significantly contribute to health‐related QoL by reducing anxiety, depression and distress, while achieving high levels of patient satisfaction (Rodriguez‐Ortega et al. 2021). BCNs are crucial in addressing patients' psychosocial needs throughout diagnosis, treatment and survivorship, providing emotional support that is often as effective as standard care (Vila et al. 2016). Research also highlights the positive impact of BCN‐led follow‐up care, with some studies indicating that telephone‐based interventions can be as effective as traditional consultations, further demonstrating the versatility of BCNs in managing complex care pathways (Liebert et al. 2003).

The involvement of BCNs enhances coordination and communication within multidisciplinary teams (MDTs), leading to a more cohesive care experience (Brown et al. 2021). The BCN model, implemented across various settings, is highly valued by both professionals and patients for navigating treatment systems and ensuring timely referrals (Liebert et al. 2003). Initially focused on supporting women with early breast cancer, the BCN role has evolved to address the needs of those with advanced breast cancer, offering a comprehensive approach that enhances patient empowerment and QoL across all stages (Brown et al. 2021; Szwajcer et al. 2004). Despite some variability in the quality of evidence, ongoing research is anticipated to provide more robust data on the effectiveness of BCNs in improving clinical outcomes and patient experiences (Rodriguez‐Ortega et al. 2021).

#### 3.3.6 Information, Communication and Educational Role

The role of BCNs is well‐established as a vital component in oncology care, with a significant positive impact on patient well‐being. BCNs, equipped with specialised training in counselling and oncology, are integral members of the multidisciplinary breast cancer team. Their ability to provide psychosocial support, education and tailored information is essential to addressing the complex needs of breast cancer patients (Rodriguez‐Ortega et al. 2021). Evidence suggests that BCNs improve the overall delivery of care, contributing to better symptom management, enhanced emotional well‐being and higher patient satisfaction (Vila et al. 2016).

Despite these benefits, gaps remain in the educational support provided to patients. Many report insufficient information on emotional changes during treatment and feel unsupported in understanding the potential psychological impacts of their diagnosis (Droog et al. 2014). This is notable, as emotional well‐being is critical to overall QoL. Studies show that BCNs who receive communication skills training are better able to identify and respond to emotional cues, improving care for patients experiencing anxiety and depression (Parle et al. 2001). Nutritional guidance is another area of concern, with many patients anxious about managing their immune health and weight gain (Droog et al. 2014). Furthermore, sexual well‐being is often neglected in patient education, with these discussions frequently absent throughout the treatment process (Droog et al. 2014; Brown et al. 2021). This oversight creates challenges in addressing the full range of psychosocial aspects impacting patients. BCNs can help bridge this gap by providing education and support on sexual health and intimacy concerns, topics that are often under‐discussed in clinical settings (Rodriguez‐Ortega et al. 2021).

To further improve patient outcomes, the educational role of BCNs must expand to provide thorough guidance on nutrition, emotional well‐being and sexual health. By addressing these gaps, BCNs can elevate care quality and enhance overall patient experiences throughout the breast cancer journey (Vila et al. 2016). Moreover, studies show that educational interventions for healthcare professionals can significantly improve their knowledge and practices related to breast cancer care. For instance, Ceber et al. (2010) demonstrated that an educational programme positively impacted nurses and midwives' knowledge of breast cancer. This highlights the importance of continuous professional development to ensure BCNs remain updated with best practices in education and care.

Additionally, a Delphi study by Drury et al. (2023) emphasised the need for specialised education programs for BCNs, particularly for advanced breast cancer. The study highlighted the need for comprehensive curricula that include clinical knowledge, person‐centred care and multidisciplinary teamwork. This reinforces the importance of continuous education for BCNs to provide tailored care that addresses the evolving needs of patients.

#### 3.3.7 Effectiveness and Evidence of BCNs Interventions

**FIGURE 2:**
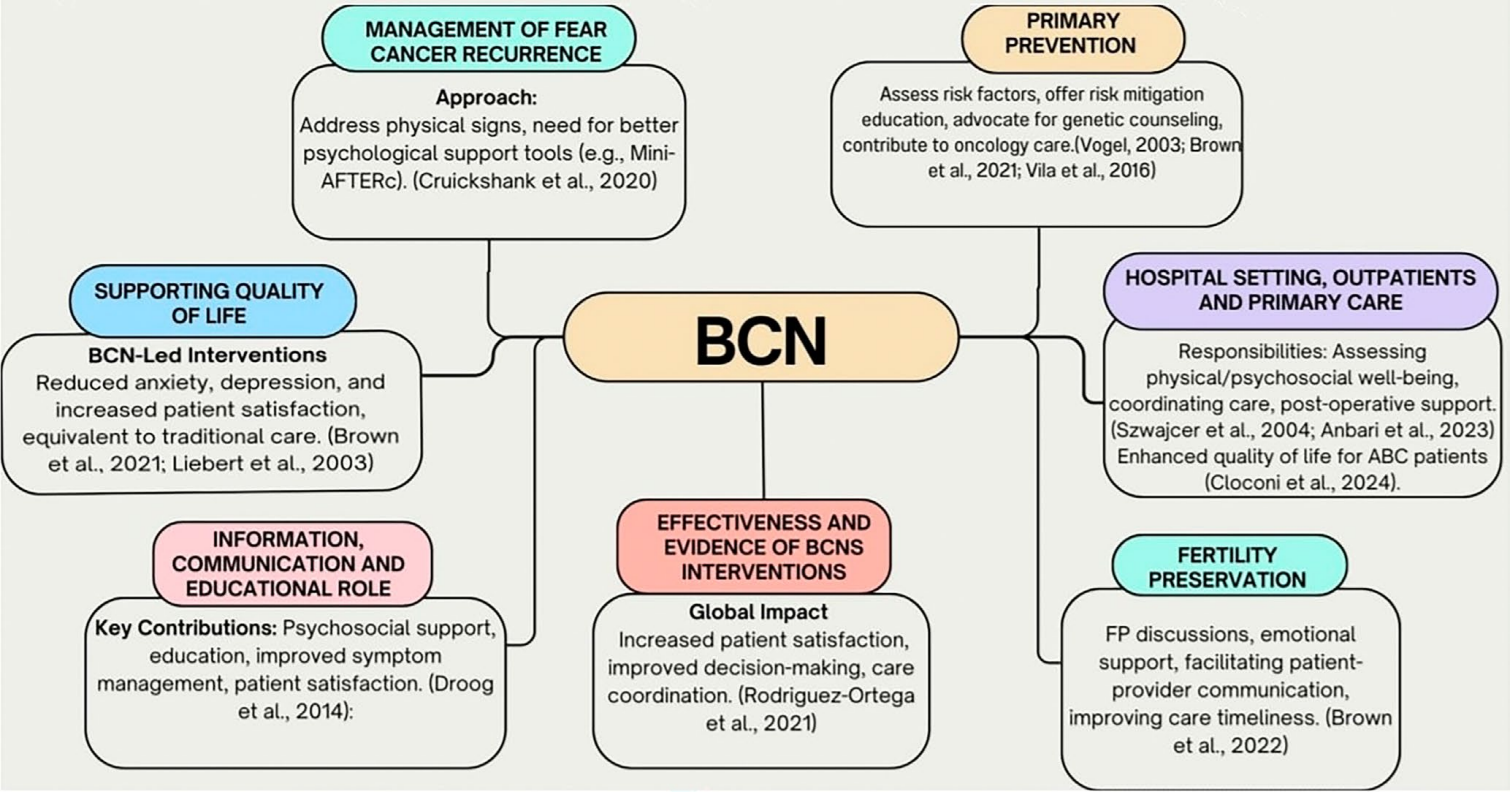
BCN's role in different settings. FP, fertility preservation.

The effectiveness of BCN interventions in breast cancer care has been demonstrated through various approaches, including psycho‐educational group interventions (PEG), individual psychological support, telephone‐based social support, home visits and presence during physician appointments. These interventions address physical, emotional and psychosocial needs, ultimately improving health outcomes for women with breast cancer (Hussain Rawther et al. 2020). Studies evaluating BCN interventions have shown reductions in emotional distress, enhancements in QoL and alleviation of treatment side effects, as well as improvements in mood, anxiety and depression management (Brown et al. 2021). Furthermore, these interventions positively impact psychological morbidity, patient satisfaction and cancer‐related worry, while also enhancing arm function and social relationships (Vila et al. 2016). BCNs provide a cost‐effective alternative to traditional care models by coordinating care and offering consistent support, promoting continuity within complex breast cancer treatment pathways (Vila et al. 2016). According to international practice guidelines and evidence reported by Rodriguez‐Ortega et al. (2021), BCNs are essential in delivering clinical expertise, educating patients and advocating for their needs. Patients with access to a BCN report higher satisfaction levels and improved communication among healthcare professionals compared to those without BCN support (Liebert et al. 2003). The presence of a BCN enhances patients' physical, psychological and social well‐being, supporting decision‐making processes and improving care coordination (Rodriguez‐Ortega et al. 2021). Personalised care that considers individual treatment history and social determinants optimises survivorship care and addresses the unique, long‐term needs of breast cancer patients (Anbari et al. 2023). Evidence supports the ongoing integration and development of the BCN role to optimise patient care, improve multidisciplinary team coordination, and meet the emotional and informational needs of breast cancer patients (Rodriguez‐Ortega et al. 2021). A graphical summary of the results of this scoping review is shown in Figure 2.

## 4 Discussion

The primary objective of this scoping review has been to identify the role, competencies and clinical areas of BCNs. This review focuses on the importance of a holistic and personalised approach, analysing the impact of integrated and collaborative care provided by specialised nurses in improving clinical outcomes and patient QoL. Additionally, it examines the post‐basic academic training pathways available for this professional role in Western contexts.

Vogel (2003) underscores the role of advanced practice nurses in the primary prevention of breast cancer, emphasising their importance in assessing risk factors, educating patients, and recommending personalised interventions such as lifestyle changes and genetic counselling. These preventative strategies can significantly reduce breast cancer incidence and mortality. In contrast, Fleure and Sara (2020) highlight the development of prostate specialist nursing roles in the UK and Australia, noting that these roles have emerged in response to distinct professional and legal frameworks. Although both regions faced challenges related to education and role definition, they have successfully implemented innovative care models emphasising holistic nursing and patient support. While Vogel (2003) calls for more research in breast cancer prevention, Fleure and Sara (2020) point to the success of prostate specialist nurses in delivering excellent care with a holistic approach.

Recent findings highlight the need to integrate prehabilitation advice and structured support into the role of BCNs, emphasising the value of guideline‐based, multidisciplinary approaches (Renouf et al. 2024). Global evidence confirms the expanding role of BCNs in addressing disparities and improving outcomes in advanced breast cancer care (Ghose et al. 2024).

Additionally, Renouf et al. (2024) highlight the importance of BCNs in delivering personalised prehabilitation advice, noting that guideline knowledge is essential for making appropriate referrals. BCNs support surveillance recommendations, pharmacotherapy, lifestyle interventions and genetic counselling, thereby improving patient outcomes (Vogel 2003). Standardising prehabilitation resources for nurses and incorporating them into referral pathways would empower BCNs and Clinical Nurse Specialists (CNSs) to provide tailored advice, enhancing patient readiness for treatment and empowering patients to make informed health decisions (Renouf et al. 2024; Vogel 2003).

The evolving managed care model presents BCNs with opportunities to enhance oncology care through patient advocacy, research and improved care outcomes (Vogel 2003). The role of CNSs in cancer care, particularly in head and neck cancer, underscores the need for substantial support throughout the treatment process. Greedy (2022) discusses how CNSs integrate expertise across various disciplines to address the wide‐ranging physical, psychological and social impacts of cancer. In comparison, Droog et al. (2014) highlight significant gaps in breast cancer care, particularly concerning nutritional needs and psychosocial aspects of the patient experience. The study points out that emotional changes, sexual well‐being and partner relationships are often neglected,an issue prevalent in oncology more broadly. This comparison underscores the importance of enhanced communication and support structures in breast cancer care, contrasting with the more coordinated approach observed in other cancer types (Pellegrinelli et al. 2024; De Pasquale et al. 2023; Greedy 2022).

The role of CNSs in prostate cryotherapy emphasises the importance of establishing comprehensive support systems for patients. Studies by Mancin et al. (2024) and Birrell and Leung (2019) describe CNSs as expert resources and advocates responsible for auditing outcomes and ensuring quality care. Their role enables autonomous decision‐making and allows for tailored, supportive follow‐up care. In contrast, Cloconi et al. (2024) highlight the involvement of BCNs in managing advanced breast cancer through radiotherapy, with a focus on palliative care and improving patients' QoL. These roles underscore the need for ongoing education and adaptation to meet the evolving needs of cancer patients, reinforcing the critical contribution of nurses in delivering comprehensive oncology care (Cloconi et al. 2024; Birrell and Leung 2019).

BCNs play a crucial role in enhancing patient outcomes, particularly in breast cancer care. Rodriguez‐Ortega et al. (2021) report that BCNs contribute significantly to patients' physical, psychological and social well‐being. They improve information provision, support decision‐making and increase patient satisfaction, although no studies have directly linked BCNs to improved survival rates. In contrast, Stewart et al. (2021) observe that lung cancer nurse specialists may occasionally enhance outcomes, though further research is needed to evaluate their long‐term impact. Both studies underscore the importance of nurse specialists in patient care, with evidence for improved clinical outcomes appearing more robust in breast cancer care (Rodriguez‐Ortega et al. 2021; Stewart et al. 2021).

Ward et al. (2023) highlight the challenges nurses encounter when supporting families affected by parental cancer, especially when children are involved. Nurses often feel underprepared to provide sufficient support, underscoring the need for education in family‐centered care, trauma‐informed approaches and child development. In the context of breast cancer care, Vila et al. (2016) emphasise the role of specialised oncology nurses within multidisciplinary teams, enhancing patients' QoL by coordinating care and providing emotional and psychological support. Collectively, these studies illustrate the need for comprehensive oncology nursing to support both patients and families throughout the cancer journey (Ward et al. 2023; Vila et al. 2016).

Nurses play a crucial role in addressing cancer‐related fatigue, especially among male patients. Robertsen and Skär (2021) highlight the importance of trust in the nurse–patient relationship, which can be cultivated by enhancing nurses' knowledge of fatigue and fostering empathetic communication. They stress the need for oncology nurses to be well informed, attentive and dedicated during patient interactions. By creating a safe environment for open dialogue, nurses can effectively support patients in managing fatigue, thus improving care quality and patient well‐being.

Being assigned a CNS significantly enhances the experience of cancer patients throughout their care journey. According to Alessy et al. (2021), CNSs improve several critical aspects of the cancer care pathway, including patient satisfaction, support and trust. Patients connected with a CNS often feel more empowered, informed and supported during their treatment. CNSs serve as a consistent point of contact, aiding patients in navigating complex healthcare systems and enhancing their overall care experience. This consistent presence underscores the vital role of CNSs in the cancer care continuum (Alessy et al. 2021).

Multidisciplinary teams recognise the pivotal role of gynaecological oncology specialist nurses in providing essential continuity and support to patients. Cook et al. (2019) describe these nurses as central figures in delivering education, conducting assessments, facilitating referrals and acting as advocates for patients. However, there are concerns about over‐reliance on these nurses, which can place excessive demands on their role. This study underscores the importance of specialist nurses in ensuring effective communication and coordination of care within the multidisciplinary team (Cook et al. 2019). Oncology nurses play a crucial role in the development and evaluation of survivorship care models, actively contributing to advancements in cancer care. Corcoran et al. (2015) emphasise how APNs significantly enhance access to quality care and improve QoL for cancer survivors. Through their roles as educators and care providers, APNs help shape survivorship care plans, supporting patients throughout their cancer journey. Bashkin et al. (2023) advocate for expanding the roles of oncology nurses to include leadership in survivorship planning and care management, thereby empowering nurses to influence the evolution of oncology practice.

In this context, the establishment of Breast Units exemplifies a critical advancement towards integrated, multidisciplinary cancer care. These units go beyond the technical aspects of breast cancer treatment, adopting a holistic approach that considers patients' psychological, social and emotional needs. This aligns with findings from Vila et al. (2016), who highlight the essential role of specialised oncology nurses in coordinating care and providing emotional support, ultimately enhancing QoL for patients. The role of BCNs within these units is multifaceted, as they leverage advanced clinical competencies to deliver personalised, patient‐centred care. By implementing primary prevention strategies, as discussed by Vogel (2003), BCNs educate patients on risk factors and lifestyle modifications, contributing to improved clinical outcomes. Moreover, the multidisciplinary nature of Breast Units fosters collaboration among healthcare professionals, a critical component for addressing the complex challenges faced by breast cancer patients, as noted by Greedy (2022). Lastly, the development of Breast Units reflects a shift towards a more collaborative, patient‐focused model of care. Within this model, the contributions of specialised nursing roles, particularly in oncology, are recognised as integral to improving health outcomes for breast cancer patients.

### 4.1 Limitations

While this review offers valuable insights into the role of BCNs, it has several limitations. A primary limitation is the variability in methodologies among the reviewed studies, which complicates direct comparisons. Additionally, the review predominantly focuses on Western healthcare settings, potentially overlooking the experiences and challenges faced by BCNs in non‐Western contexts. This geographic limitation may restrict the generalisability of the findings. Moreover, further research is needed to assess the long‐term impact of BCN interventions on patient survival and QoL.

### 4.2 Future Directions for Research

The role of BCNs is set to evolve as healthcare systems increasingly prioritise holistic and personalised care. BCNs are anticipated to play a leading role in developing patient‐centred care models that integrate risk assessments, education and tailored interventions (Vogel 2003). Future research should aim to standardise metrics for assessing BCN impact on patient outcomes, including QoL and survivorship care. Longitudinal studies are crucial to evaluate the long‐term effects of BCN interventions on clinical outcomes and patient satisfaction. Additionally, exploring the role of BCNs across diverse healthcare systems will provide a more comprehensive view of their contributions to global cancer care. Research should also investigate how technological advancements, such as telehealth, can enhance BCN practices and support (Renouf et al. 2024).

## 5 Conclusions

BCNs are essential in delivering comprehensive care to breast cancer patients, particularly those with advanced disease. Through specialised education, care coordination and psychosocial support, BCNs contribute to improved clinical outcomes and enhanced QoL for patients. Their role is especially valuable in addressing unmet needs, such as fertility preservation, emotional support and managing fear of cancer recurrence. While the positive impact of BCN interventions is well documented, ongoing professional development and expanded educational programmes are vital to meet the evolving needs of patients. Strengthening these areas will further empower BCNs to provide personalised and holistic care.

### 5.1 Relevance to Clinical Practice

Integrating BCNs into multidisciplinary teams has profound implications for clinical practice and nursing policy. Vila et al. (2016) emphasise that BCNs streamline care transitions and provide educational resources that empower patients throughout their cancer journey. Their role enhances patient satisfaction and adherence to treatment plans. Alessy et al. (2021) also emphasise that BCNs can improve patient experiences by serving as vital links between patients and healthcare teams. Nursing policy should advocate for the recognition and standardisation of the BCN role, ensuring adequate training and resources to support these practitioners. Expanding oncology nurses' competencies to include leadership in survivorship planning is also essential to meet the growing needs of cancer survivors (Corcoran et al. 2015).

## Author Contributions

**Lucia D'Alessandro:** conceptualization, methodology, writing original draft, review and editing, investigation, visualisation; **Sara Morales Palomares:** writing original draft, review and editing, investigation; **Stefano Mancin:** writing original draft, review and editing, investigation, coordinator; **Marco Sguanci:** methodology, review and editing, investigation, visualisation; **Daniela Cattani:** review and editing, visualisation; **Simone Cosmai:** review and editing, visualisation; **Diego Lopane:** review and editing, visualisation; **Margarita Gjeloshi:** review and editing, visualisation; **Giovanni Cangelosi:** review and editing, visualisation, coordinator; **Beatrice Mazzoleni:** review and editing, investigation, visualisation, coordinator. Lucia D'Alessandro and Sara Morales Palomares provided an equal contribution as first author in drafting the manuscript; Giovanni Cangelosi and Beatrice Mazzoleni provided an equal contribution as last author to the coordination of the research group. All authors read and approved the final manuscript.

## Disclosure

The scoping review protocol was prospectively registered on the Open Science Framework, available at: 10.17605/OSF.IO/DU82V.

## Conflicts of Interest

The authors declare no conflicts of interest.

## Supporting information


Data S1.

Table S1.

## Data Availability

Data sharing is not applicable to this article as no new data were created or analyzed in this study.
