# Supplementary material for: Professional Roles, Skills and Advanced Educational Programs of Breast Cancer Nurses: A Scoping Review
Source: Nurs Open. 2025 Sep 28;12(10):e70251. doi: 10.1002/nop2.70251 (PMC12476883; doi:10.1002/nop2.70251)
Supplement: Supplementary file 2 — Table S1. [file NOP2-12-e70251-s001.docx]

**TABLE S1.** Search strategy

| **ID** | **Cochrane Library** | **Number of articlesfound** |
| --- | --- | --- |
| #1 | “Clinical Nurse Specialist” OR “Advanced Practice Nurse” OR “Clinical Nurse Consultant” OR “Nurse Practitioner” OR “Advanced Practice Registered Nurse” OR “Specialist Nurse Practitioner” OR “Clinical Nursing Specialist” OR “Advanced Clinical Nurse” OR “Clinical Nurse Leader” OR “Clinical Nurse Educator” OR “Clinical Advanced Practice Nurse” OR “Clinical Nursing Consultant” | **Results** |
| #2 | "Breast Neoplasm" OR "Neoplasms Breast" OR "Breast Tumors" OR "Breast Tumor" OR "Tumor Breast" OR "Tumors Breast" OR "Breast Cancer" OR "Cancer Breast" OR "Cancer of Breast" OR "Cancer of the Breast" OR "Malignant Neoplasm of Breast" OR "Breast Malignant Neoplasm" OR "Breast Malignant Neoplasms" OR "Malignant Tumor of Breast" OR "Breast Malignant Tumor" OR "Breast Malignant Tumors" OR "Mammary Cancer" OR "Cancer Mammary" OR "Cancers Mammary" OR "Mammary Cancers" OR "Mammary Neoplasms Human" OR "Human Mammary Neoplasm" OR "Human Mammary Neoplasms" OR "Neoplasm Human Mammary" OR "Neoplasms Human Mammary" OR "Mammary Neoplasm Human" OR "Breast Carcinoma" OR "Breast Carcinomas" OR "Carcinoma Breast" OR "Carcinomas Breast" OR "Mammary Carcinoma Human" OR "Carcinoma Human Mammary" OR "Carcinomas Human Mammary" OR "Human Mammary Carcinomas" OR "Mammary Carcinomas Human" OR "Human Mammary Carcinoma" |  |
| **#3** | **#1 AND #2** | **71** |
| **ID** | **PubMed** | **Results** |
| #1 | "Nurse Specialists"[Mesh] OR “Clinical Nurse Specialist” OR “Advanced Practice Nurse” OR “Clinical Nurse Consultant” OR “Nurse Practitioner” OR “Advanced Practice Registered Nurse” OR “Specialist Nurse Practitioner” OR “Clinical Nursing Specialist” OR “Advanced Clinical Nurse” OR “Clinical Nurse Leader” OR “Clinical Nurse Educator” OR “Clinical Advanced Practice Nurse” OR “Clinical Nursing Consultant” |  |
| #2 | "Breast Neoplasms"[Mesh] OR "Breast Neoplasm" OR "Neoplasms Breast" OR "Breast Tumors" OR "Breast Tumor" OR "Tumor Breast" OR "Tumors Breast" OR "Breast Cancer" OR "Cancer Breast" OR "Cancer of Breast" OR "Cancer of the Breast" OR "Malignant Neoplasm of Breast" OR "Breast Malignant Neoplasm" OR "Breast Malignant Neoplasms" OR "Malignant Tumor of Breast" OR "Breast Malignant Tumor" OR "Breast Malignant Tumors" OR "Mammary Cancer" OR "Cancer Mammary" OR "Cancers Mammary" OR "Mammary Cancers" OR "Mammary Neoplasms Human" OR "Human Mammary Neoplasm" OR "Human Mammary Neoplasms" OR "Neoplasm Human Mammary" OR "Neoplasms Human Mammary" OR "Mammary Neoplasm Human" OR "Breast Carcinoma" OR "Breast Carcinomas" OR "Carcinoma Breast" OR "Carcinomas Breast" OR "Mammary Carcinoma Human" OR "Carcinoma Human Mammary" OR "Carcinomas Human Mammary" OR "Human Mammary Carcinomas" OR "Mammary Carcinomas Human" OR "Human Mammary Carcinoma" |  |
| **#3** | **#1 AND #2** | **343** |
| **ID** | **Cumulative Index of Nursing and Allied Health Literature (CINAHL)** | **Results** |
| #1 | “Clinical Nurse Specialist” OR “Advanced Practice Nurse” OR “Clinical Nurse Consultant” OR “Nurse Practitioner” OR “Advanced Practice Registered Nurse” OR “Specialist Nurse Practitioner” OR “Clinical Nursing Specialist” OR “Advanced Clinical Nurse” OR “Clinical Nurse Leader” OR “Clinical Nurse Educator” OR “Clinical Advanced Practice Nurse” OR “Clinical Nursing Consultant” |  |
| #2 | "Breast Neoplasm" OR "Neoplasms Breast" OR "Breast Tumors" OR "Breast Tumor" OR "Tumor Breast" OR "Tumors Breast" OR "Breast Cancer" OR "Cancer Breast" OR "Cancer of Breast" OR "Cancer of the Breast" OR "Malignant Neoplasm of Breast" OR "Breast Malignant Neoplasm" OR "Breast Malignant Neoplasms" OR "Malignant Tumor of Breast" OR "Breast Malignant Tumor" OR "Breast Malignant Tumors" OR "Mammary Cancer" OR "Cancer Mammary" OR "Cancers Mammary" OR "Mammary Cancers" OR "Mammary Neoplasms Human" OR "Human Mammary Neoplasm" OR "Human Mammary Neoplasms" OR "Neoplasm Human Mammary" OR "Neoplasms Human Mammary" OR "Mammary Neoplasm Human" OR "Breast Carcinoma" OR "Breast Carcinomas" OR "Carcinoma Breast" OR "Carcinomas Breast" OR "Mammary Carcinoma Human" OR "Carcinoma Human Mammary" OR "Carcinomas Human Mammary" OR "Human Mammary Carcinomas" OR "Mammary Carcinomas Human" OR "Human Mammary Carcinoma" |  |
| **#3** | **#1 AND #2** | **201** |
| **ID** | **EMBASE** | **Results** |
| #1 | 'clinical nurse specialist':ab,ti OR 'advanced practice nurse':ab,ti OR 'clinical nurse consultant':ab,ti OR 'nurse practitioner':ab,ti OR 'advanced practice registered nurse':ab,ti OR 'specialist nurse practitioner':ab,ti OR 'clinical nursing specialist':ab,ti OR 'advanced clinical nurse':ab,ti OR 'clinical nurse leader':ab,ti OR 'clinical nurse educator':ab,ti OR 'clinical advanced practice nurse':ab,ti OR 'clinical nursing consultant':ab,ti |  |
| #2 | "Breast Neoplasm" OR "Neoplasms Breast" OR "Breast Tumors" OR "Breast Tumor" OR "Tumor Breast" OR "Tumors Breast" OR "Breast Cancer" OR "Cancer Breast" OR "Cancer of Breast" OR "Cancer of the Breast" OR "Malignant Neoplasm of Breast" OR "Breast Malignant Neoplasm" OR "Breast Malignant Neoplasms" OR "Malignant Tumor of Breast" OR "Breast Malignant Tumor" OR "Breast Malignant Tumors" OR "Mammary Cancer" OR "Cancer Mammary" OR "Cancers Mammary" OR "Mammary Cancers" OR "Mammary Neoplasms Human" OR "Human Mammary Neoplasm" OR "Human Mammary Neoplasms" OR "Neoplasm Human Mammary" OR "Neoplasms Human Mammary" OR "Mammary Neoplasm Human" OR "Breast Carcinoma" OR "Breast Carcinomas" OR "Carcinoma Breast" OR "Carcinomas Breast" OR "Mammary Carcinoma Human" OR "Carcinoma Human Mammary" OR "Carcinomas Human Mammary" OR "Human Mammary Carcinomas" OR "Mammary Carcinomas Human" OR "Human Mammary Carcinoma" |  |
| **#3** | **#1 AND #2** | **866** |
| **TOTAL ARTICLES FOUND** | | **1481** |
